# Semen problem in beta-thalassemia: an interesting focus on tropical reproductive science and hematology

**Published:** 2012-09

**Authors:** Viroj Wiwanitkit

**Affiliations:** *Wiwanitkit House, Bangkhae, Bangkok, Thailand.*

Dear Editor,

The problem of congenital hemoglobin disorder is common in tropical Asia. In tropical Southeast Asian countries, very high prevalence of thalassemia disorder especially for beta thalassemia is observed. This tropical hematological problem affects millions of population and cause several health disorders. Of interest, the issue of reproductive health impairment of the population with beta thalassemia disorder is not well mentioned. 

Here, the author tried to summarize some important information on the semen problem in beta-thalassemia. In beta-thalassemia major, poor semen quality is observed. It is noted that most patients have “hypogonadotropic hypogonadism state, impairment fertility and growth retardation (1).” Jensen *et al* proposed that “The causation is multi-factorial, with iron deposition in the pituitary gland resulting from life-long dependence on blood transfusions being a major factor (2).”

It is evidenced that the sperm DNA damage is due to oxidative stress from iron overload (3-4). Nevertheless, it is reported that the spermatogenesis in the patients are also suppressed by chelation therapy, desferrioxamine (5). The degree of sperm DNA damage is interestingly high in the case with low ferritin level (5-6). It seems that the exacts mechanism of semen problem in beta thalassemia patient is very complex and relating to unknown disturbance of iron metabolism impairment in the patients.
